# Research and Analysis of the Propagation of Vertical Vibrations in the Arrangement of a Vehicle Seat—A Child’s Seat

**DOI:** 10.3390/s21248230

**Published:** 2021-12-09

**Authors:** Andrzej Zuska, Damian Frej, Jerzy Jackowski, Marcin Żmuda

**Affiliations:** 1 Department of Automotive Engineering and Transport, Kielce University of Technology, Avenue Tysiaclecia Państwa Polskiego 7, 25-314 Kielce, Poland; a.zuska@tu.kielce.pl; 2 Department of Mechanical Engineering, Institute of Vehicles and Transportation, Military University of Technology (WAT), ul. gen. Sylwestra Kaliskiego 2, 00-908 Warsaw, Poland; jerzy.jackowski@wat.edu.pl (J.J.); marcin.zmuda@wat.edu.pl (M.Ż.)

**Keywords:** vehicle dynamics, vibrations, vehicle safety, vehicle passive safety, vehicle testing

## Abstract

This paper deals with the issues of the impact of vertical vibrations on a child seated in a child seat during a journey. Its purpose was to assess the impact of fastening the child seats and road conditions on the level of vibrations recorded on child seats. The paper describes the tested child seats, the methodology of the tests and the test apparatus included in the measuring track. The tests were carried out in real road conditions where the child seats were located on the rear seat of a passenger vehicle. One was attached with standard seat belts, and the other with the ISOFIX base. When driving on roads with three types of surface, the following vertical accelerations were measured: seat of the child seats, the rear seat of the vehicle and the ISOfix base. The recorded accelerations were first analyzed in the time domain and then in the frequency domain. Three indexes (r.m.s, rmq and VDV) were used to assess the vibration comfort. Research has shown that the classic method of fastening a child seat with standard seat belts is more advantageous in terms of vibration comfort. Calculated indicators confirmed the negative impact of separating the child seat from the rear seat of the vehicle using the IQ ISOFIX base.

## 1. Introduction

In Poland and in other European Union countries, transporting children in child seats is obligatory. The main task of child seats is to ensure the child’s safety and reduce any possible injuries that may occur during a collision or road accident [1,2,3,4,5,6,7,8]. There are many design solutions for child seats on the market, which differ in terms of the fastening system, construction material and safety level, and selecting the ideal seat is a difficult task for buyers [9,10,11,12,13,14]. Immediately after buying the child seat, there is a problem with its installation in the vehicle. It is estimated that in Poland and Union European approximately 70% of child seats are installed incorrectly [15,16,17]. The most common mistakes when mounting the seat in the vehicle include choosing the wrong place for its installation, loose mounting, an incorrectly adjusted headrest, and incorrectly defined seat angle [16,17,18]. An improperly selected and, in addition, a badly installed child seat, instead of protecting the child against the effects of collisions and road accidents, may be an additional risk for the child [19,20,21]. The first child seats were constructed at the turn of the 1920s and 1930s [22]. Initially, their main task was to keep the child in a specific place, and not to ensure adequate safety [23,24]. Gradually, the development of child seats was equated with the knowledge concerning the behavior of the human body during road accidents. A breakthrough in the design of child seats occurred in 1966, when Bertil Aldman, a Doctor of Medical Sciences, published a research paper in which he pointed to the advantages of rear-facing child seats mounted on the front seat [4,22,25]. A year later, they were put on sale. Moreover, for the first time, they were also equipped with safety belts intended for children [22,23,25].

Increasingly, sensors are installed in child seats, which check the correctness of their fastening [25,26,27]. The child seats available on the market have many technical solutions, the main purpose of which is to increase the safety level of children while traveling. These solutions include [5,7]:

- ISOFIX—mounting system,
- a stabilizing leg,
- top fastening belt,
- side protection system,
- five-point seat belts.

Designers of child seats pay special attention to the comfort of children when travelling [25,26,27]. Manufactured child seats equipped with temperature sensors already exist, which provide information about whether the temperature in the vehicle is appropriate for the child being transported. The child seat prototype proposed by Mercedes-Benz and Britax Römer has a child behavior monitoring system. It records, among other things, a child’s body temperature, the number of breaths, as well as whether the child is sleeping or not. This information is provided to the child’s caregiver and thus reduces the possibility of distraction posed by constantly looking at the transported child. The introduction of new technologies to the production of child seats have been motivated by the necessity to look for solutions that may increase mobility and reduce driver distraction while driving [5,28,29].

UNECE Regulation No. 44 includes the division of child seats according to their body weight [30,31,32]:

- group 0 (0+)—child seats for children mass to 10 kg (13 kg),
- group I—child seats for children mass from 9 kg to 18 kg,
- group II—child seats for children mass from 15 kg to 25 kg,
- group III—child seats for children mass from 22 kg to 36 kg.

The proper selection of a child seat is related not only to its age but also to its anthropometric dimensions, such as mass and height [33,34]. Considering only one of the above-mentioned sizes in the seat selection is insufficient. It is also worth paying attention to the possibility of adjusting the height of the backrest and the width of the seat. A child in a child seat should first feel comfortable and safe [18,35,36].

The subject of vibration comfort of adult motor vehicle users, as opposed to the issue of the vibration comfort of children transported in child seats, has been quite thoroughly explored. The comfort felt by a child differs to a large extent from that of an adult [37,38,39,40,41]. In addition, an adult can correctly determine the degree of perceived comfort, while in the case of a small child it is impossible. The issues of vibrational comfort of adults have been discussed in many normative documents [42,43,44] The standards also describe the measures of the influence of vertical vibrations on a person in a sitting position There are no such standards for children in child seats [42,43,44,45,46,47]. The development of safety seats should go hand in hand with the development of vibration comfort [48,49,50]. The study in [51] presents the results of vibration tests carried out with the use of two child seats while driving on roads with different surfaces.

The results showed that the vibrations measured on the seat of the child seat turned out to be higher than the vibration level on the driver’s seat. Similar conclusions were presented in the study in [52]. The influence of wheel unbalance on the vibration comfort of children was discussed in [53]. The result of the ergonomic comfort tests, during which the distribution of pressure of the child’s body on the seat and backrest of child seats was analyzed, was a product-design assessment model (PDE) that facilitates the selection of materials and the angle of the backrest for manufacturers of child seats [54]. The result of this research is a model that makes it easier for child seat manufacturers to choose materials and tilt the backrests [51,52,53]. When reviewing the literature, it should be noted that child seats are mainly tested in terms of safety. Vibration comfort issues are unfortunately overlooked.

Importantly, it should be noted that despite the importance of the vibration comfort of child seats, it is neglected, and there are no child seats on the market that are equipped with acceleration sensors, which would allow the assessment of vibration comfort. However, child seats currently available on the market are becoming more and more advanced. Vehicle seats with sensors informing whether the child has properly fastened seat belts now exists, as well as an ISOFIX base with a sensor that provides information about the correct fastening of the seat [50,51,52,53,54]. It should be noted, however, that there are solutions for child seats, which are additionally equipped with a temperature sensor and sensors that allow for blood pressure measurements [55,56,57,58,59,60], Equipping safety seats with various types of sensors increases ergonomic comfort and improves safety.

## 2. Research Methodology

The aim of the experiment was to analyze the impact of road conditions, the child’s mass, and the method of mounting child seats on the vibration comfort of the children transported in it. The research was carried out on three sections of roads with the following surfaces: asphalt, gravel, and paving. A passenger vehicle of a lower-middle class was used in the research. To limit the influence of the technical condition of the test car on the measurement results, the vehicle was subjected to additional technical tests before the initiation of the tests. When inspecting and assessing the technical condition of the vehicle, particular attention was paid to the suspension and running gear. Among other things, the technical condition of the shock absorbers was reviewed, as well as the correctness of wheel balancing and the air pressure in the tires. During the experiment, the air pressure in the tires was 0.25 MPa in the front axle wheels and 0.2 MPa in the rear axle wheels, respectively. The wheels were balanced with an accuracy of 2 g.

During the tests, two child seats were attached to the rear seat of the test vehicle. For one of them, an Avionaut Pixel was attached in the standard way with the vehicles seat belts, while for the other an Avionaut AeroFIX was fastened with the ISOFIX system.

Three series of studies were conducted. Within each of them, three vehicle rides with mounted child seats were conducted. During each of the journeys, the child seats were loaded with a different mass imitating the mass of a child of 5 kg, 10 kg, and 15 kg, respectively. The speed of the vehicle during each trip was maintained at a constant level of 50 km/h.

## 3. Research Object

**Figure 1:**
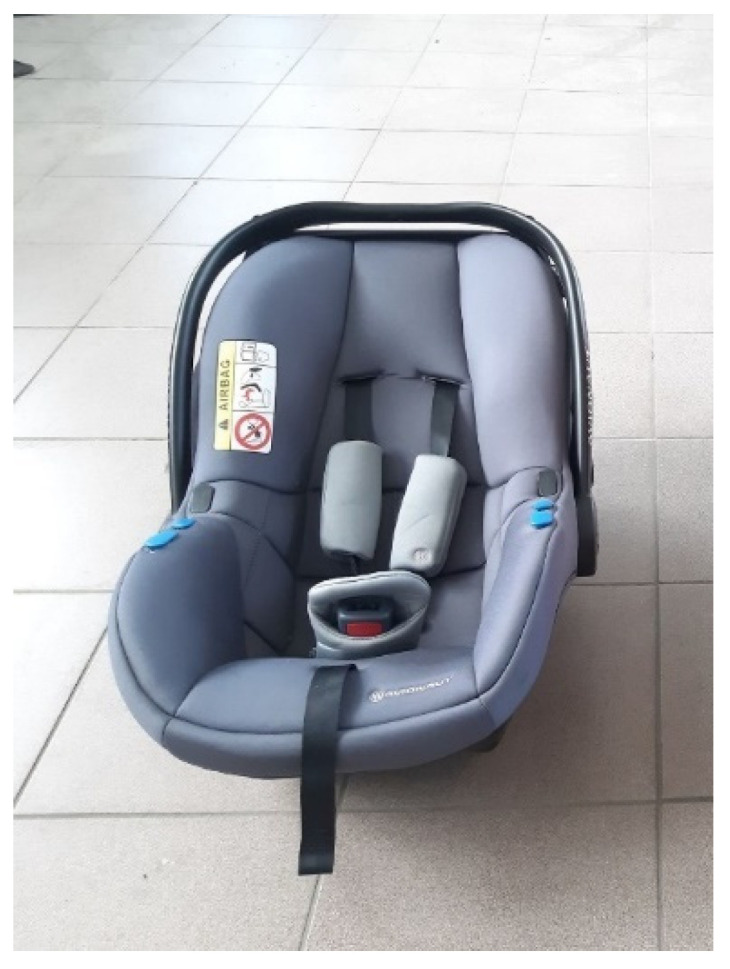
Avionaut Pixel child seat.

**Figure 2:**
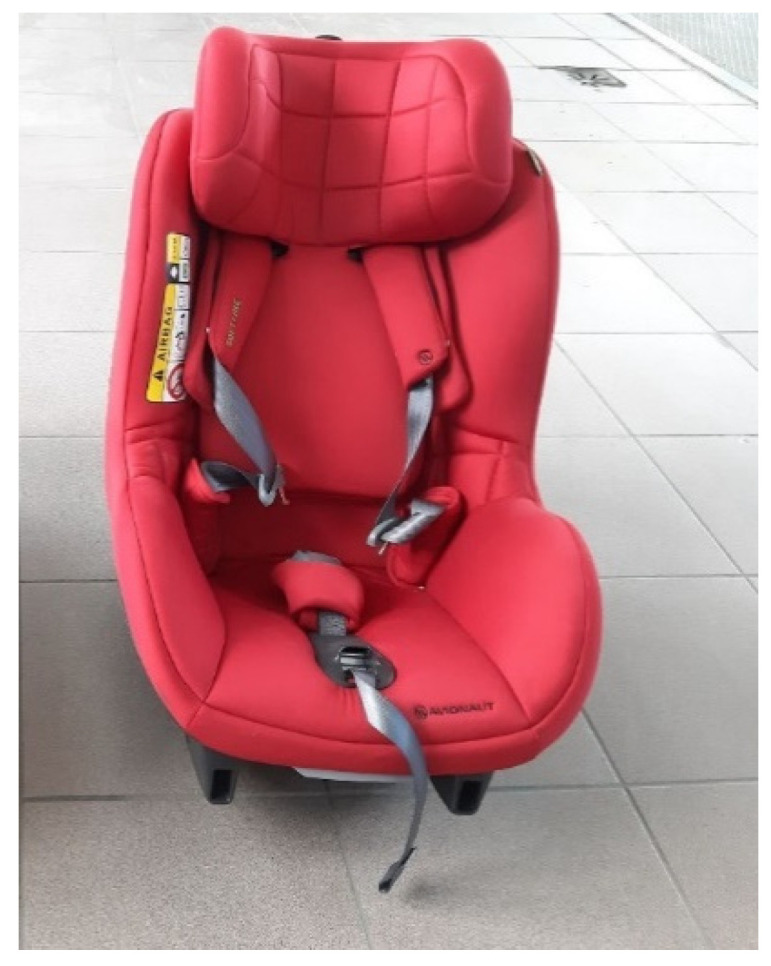
Avionaut AeroFIX child seat.

The following two seats were used in the experiment: Avionaut Pixel (Figure 1) and Avionaut AeroFIX (Figure 2). The technical specifications of the child seats are presented in Table 1.

The Avionaut Pixel child seat is designed to transport children mass up to 13 kg and a height from 0.45 m to 0.86 m. Its curb mass is 2.5 kg. It is made of a composite material with an EPP ARPRO designation, which does not deform during impact, but due to its flexibility it absorbs energy, distributing it evenly throughout its structure [61].

The Avionaut AeroFIX seat is designed to transport children with a height of 0.67 m to 1.05 m and a mass of up to 17.5 kg. It has been designed in such a way that it is possible to transport a child in a rearward-facing position, if their height does not exceed 1.05 m. Its curb weight is 4 kg. The child seat has a side protection system that protects the child in side impacts. The child seat was attached to a dedicated device, called the ISOFIX base. It allows for the seat to be mounted in the vehicle both forward and rearward facing. In vehicles not equipped with this type of system, both child seats can be fastened with the seat belts [61].

## 4. Research Apparatus

During the experiment, the longitudinal speed of the vehicle was controlled by a measuring system consisting of an optoelectronic sensor of longitudinal and lateral speed (Correvit S-350), a data acquisition system and computer. The sensor was attached to the body of the test vehicle by means of a holder, which allowed for the adjustment of the position of the sensor in relation to the road surface. The built-in measuring system registered the speed in the range from 0.5 km/h to 250 km/h, and this signal can be updated with a frequency of up to 250 Hz.

**Figure 3:**
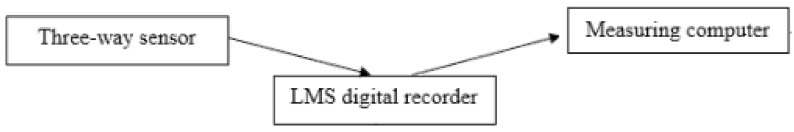
Diagram of the measurement path.

In the research, the acceleration of rear seats was measured with ISOfix bases, Avionaut Pixel seats, and Avionaut AeroFIX seats. The accelerations were recorded using a measurement track (Figure 3) consisting of four three-way acceleration sensors (Table 2), a digital LMS SCADAS Recorder model SCR02 and a measuring computer. The frequency of recording the acceleration signals was 1024 Hz. The measuring line was powered by a 12 V battery with the use of a converter.

**Figure 4:**
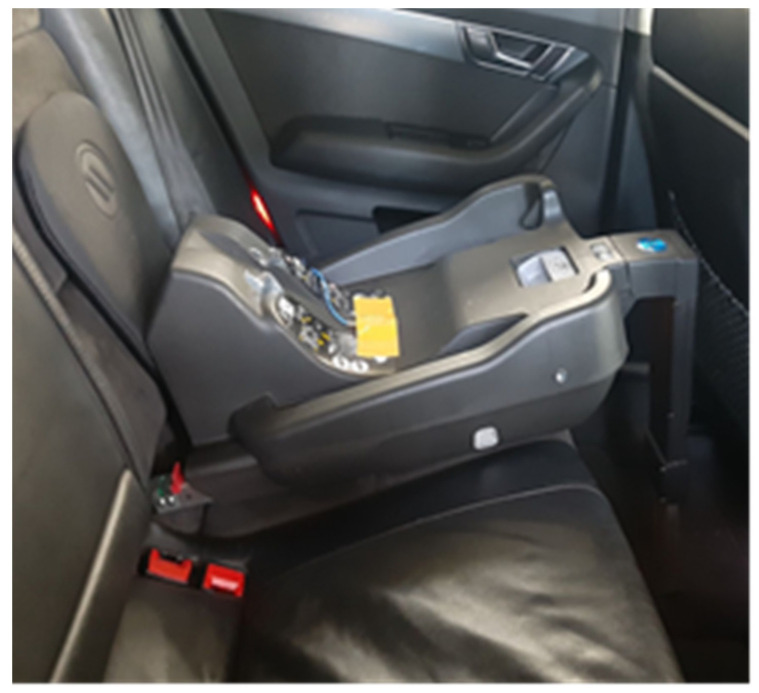
Mounting the acceleration sensor on the Isofix base.

**Figure 5:**
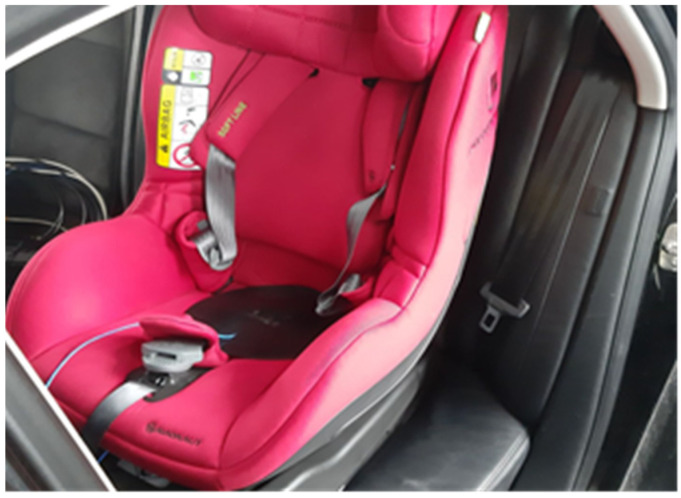
Fastening the acceleration sensor to the child seat.

**Figure 6:**
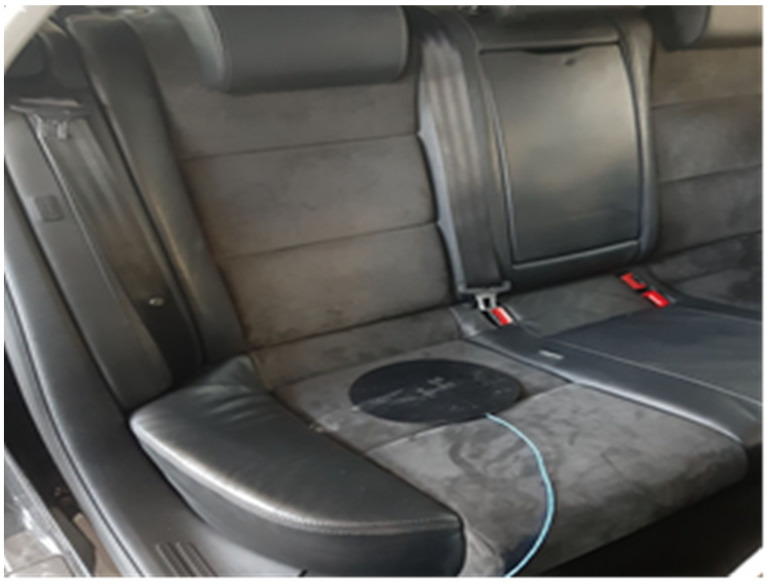
Fixing the acceleration sensor to the rear seat.

**Figure 7:**
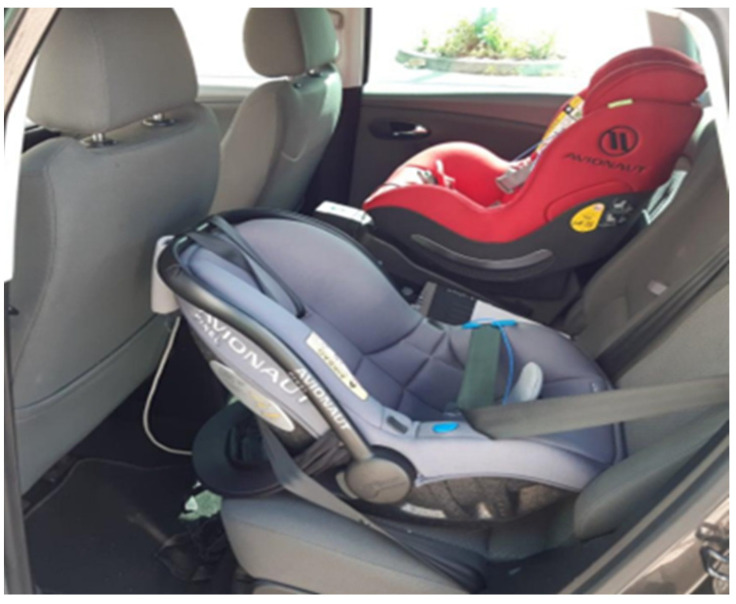
Fixing child seats in a passenger vehicle.

Places showing the mounting points of the acceleration sensors are shown in Figure 4, Figure 5 and Figure 6, and the method of their mounting is shown in Figure 7.

## 5. Road Test Results and Their Analysis

**Figure 8:**
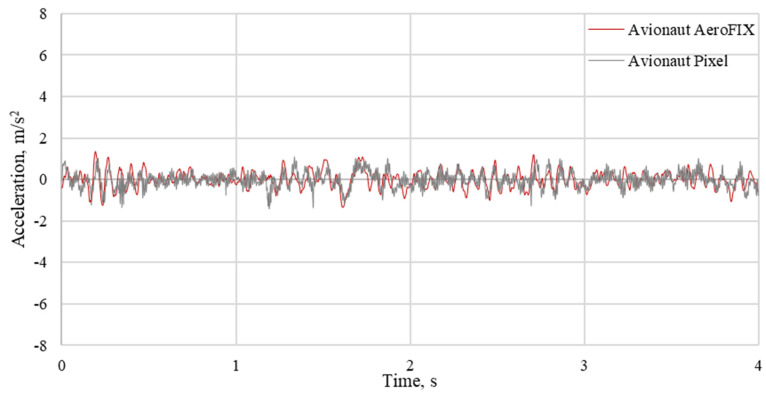
The vertical accelerations recorded on the seat of child seats loaded with a mass of 10 kg driving on an asphalt road.

**Figure 9:**
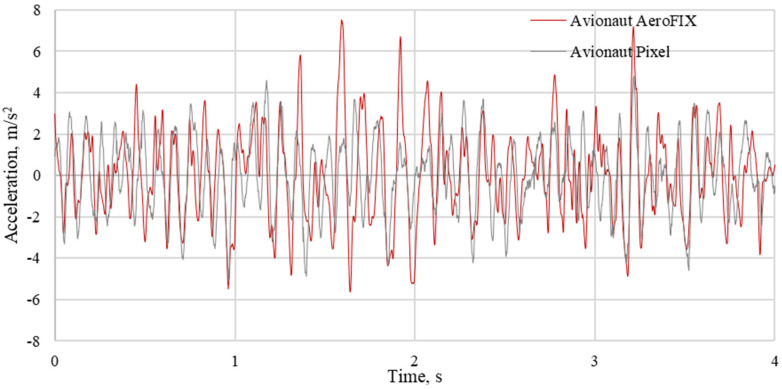
Vertical accelerations recorded on the seat of child seats loaded with a mass of 10 kg when driving on a gravel road.

**Figure 10:**
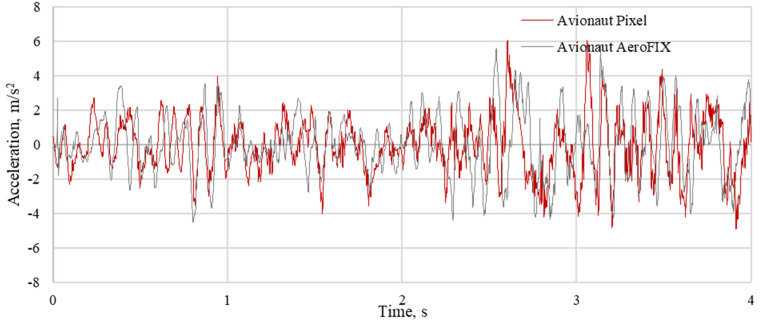
The vertical accelerations recorded on the seat of the child seats weighed 10 kg when driving on a cobblestone road.

The results of the measurements were used to determine the time courses of vertical accelerations and indicators for the assessment of vibration comfort. Examples of waveforms of vibrations recorded on the seats of the tested child seats are shown in Figure 8, Figure 9 and Figure 10. The recorded courses of acceleration for the seat of the AvionautPixel child seat and the seat of the AvionautAeroFIX child seat are similar in terms of quality. However, there are quantitative differences between them. In each of the analyzed journeys, higher acceleration values were recorded on the seat of the Avionaut AeroFIX child seat.

Considering the fact that vibrations are quite a complex phenomenon, it is impossible to analyze them comprehensively using only one measure. With the directly measured vibration accelerations, comfort indicators were used to assess their impact on the vibration comfort. These indicators describe the ISO 2631-1 and BS 6841 standards. Depending on the acceleration value m.s, ISO 2631-1: 1997 specifies the level of comfort on a six-point scale. The same discomfort scale with regard to rmq is found in BS 6841. In this analysis, the authors decided to use the following three measures:

- Root mean square (rms)
  (1) $$r.m.s={\left[\frac{1}{T}\int_{0}^{T}a^{2}\left(t\right)\mathrm{dt}\right]}^{\frac{1}{2}}$$
- Vibration Dose Value (VDV)
  (2) $$\mathrm{VDV}={\left[\int_{0}^{T}a^{4}\left(t\right)\mathrm{dt}\right]}^{\frac{1}{4}}$$
- Root mean quad (rmq)
  (3) $$\mathrm{rmq}={\left[\frac{1}{T}\int_{0}^{T}a^{4}\left(t\right)\mathrm{dt}\right]}^{\frac{1}{4}}$$

where:

a (t)—vertical acceleration value, m/s^2^, recorded as a function of time t
T—segment of the measurement duration, s.

Table 3 presents the summary of the values of the determined indicators of the vibration comfort assessment rms, VDV and rmq for the measurements carried out with the child seats loaded with a mass of 5 kg, 10 kg, and 15 kg.

**Figure 11:**
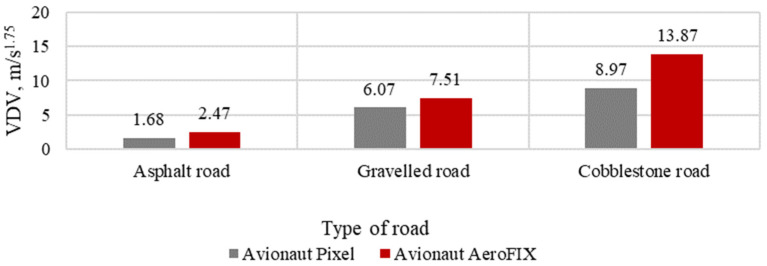
VDV indicator with a child seat load of 5 kg.

**Figure 12:**
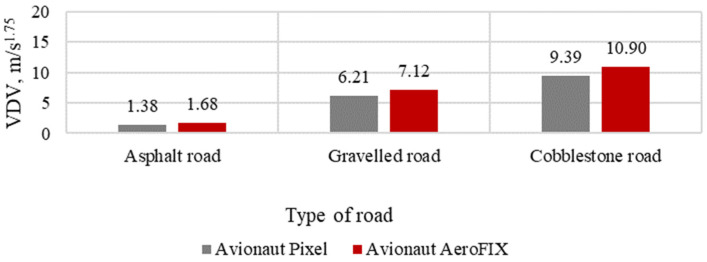
VDV indicator with a child seat load of 10 kg.

**Figure 13:**
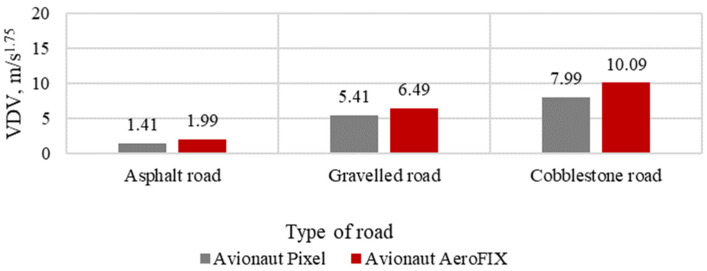
VDV indicator with a child seat load of 15 kg.

The results of the assessment of vibration comfort, according to the VDV index for the tested seats, are presented in Figure 11, Figure 12 and Figure 13.

**Figure 14:**
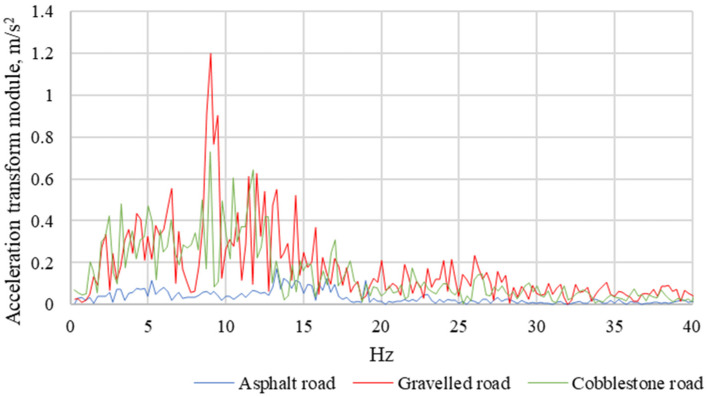
Amplitude-frequency characteristics of the seat of the Avionaut AeroFIX child seat with a mass of 10 kg.

**Figure 15:**
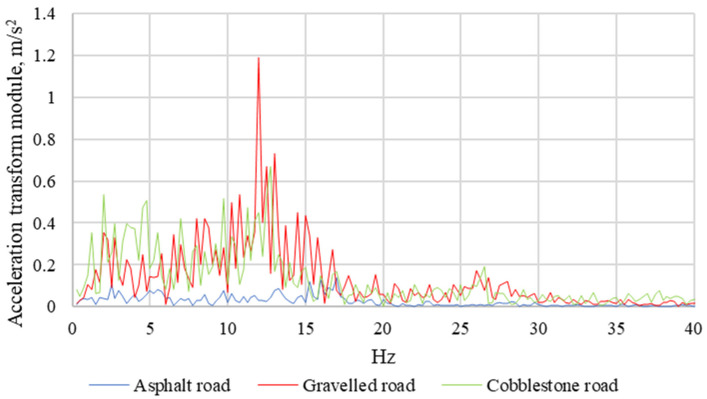
Amplitude-frequency characteristics of the seat of the Avionaut Pixel child seat with a mass of 10 kg.

The correct fastening of the child seat and the materials from which the child seats are made are important to dampen vibrations that penetrate from the road surface to the child seat. In the next stage of the analysis, each of the recorded signals was subjected to a discrete Fourier transformation and then the amplitude-frequency characteristics were determined. Their examples are shown in Figure 14 and Figure 15.

## 6. Analysis of the Results

The analysis of the rms values of accelerations listed in Table 3 shows that the accelerations recorded on the child seat fastened with seat belts are lower than the accelerations recorded on the seat fastened with the IQISOFIX base. The type of road surface on which the tests were carried out had a significant impact on the level of acceleration, apart from the method of mounting and the mass loading the seat.

The lowest acceleration values were recorded for the seats of the child seats during journeys made on an asphalt road. The RMS values for these accelerations for the Avionaut Pixel child seat range from 0.34 m/s^2^ to 0.4 m/s^2^. They are lower than the effective rms values of acceleration recorded on the Avionaut AeroFIX child seat, which are in the range from 0.39 m/s^2^ to 0.62 m/s^2^.

The highest accelerations were recorded while driving on the paved surface on the seat of the child seat. The effective rms values for these accelerations for the Avionaut Pixel child seat range from 2.18 m/s^2^ to 2.58 m/s^2^. They are lower than the effective rms values of acceleration recorded on the Avionaut AeroFIX child seat, which are in a range from 2.69 m/s^2^ to 3.56 m/s^2^.

Relating the determined effective values of rms accelerations to the limits of discomfort of adults, it should be stated that: Transporting children in the Avionaut Pixel child seat on the asphalt and gravel road may cause them slight discomfort, while on the paved road, the child may feel very or extremely uncomfortable. Transporting children in the Avionaut AeroFIX child seat on an asphalt road and in one case (when its mass is 15 kg) on a gravel surface may cause them a feeling of slight discomfort. In other cases, the child transported in this child seat may feel very or extremely uncomfortable.

The rqm indicator provides a greater share in the assessment of accelerations with higher amplitudes, due to the fourth power of accelerations. It is more sensitive to acceleration pulses. This effect can be observed by comparing the values of the rms and rqm indicators, determined for the acceleration curves recorded during runs on the asphalt road with the rms and rqm indicators determined for the acceleration curves recorded during the runs on gravel and paved roads. The highest values of the rmq index were recorded for the runs on the gravel surface. This is due, inter alia, to the fact that the surface was covered with numerous irregularities.

For the determined amplitude-frequency, characteristics bands of local maxima were observed. The acceleration amplitudes are the highest when driving on a gravel road. Being in the range from 5 Hz to 15 Hz, they are ten times higher than the acceleration amplitudes recorded during asphalt road journeys and over 35 percent higher than the acceleration amplitudes recorded during paved-road journeys.

## 7. Conclusions

For a child seat to be approved for sale, it must pass a series of safety tests. Currently, seats that meet the requirements of ECE R44 and UN R129 are approved for sale. It should be noted, however, that child seats approved for sale are not tested in terms of comfort. Unfortunately, the omission of such an important factor in the production of child seats results in vibrations generated by road unevenness being largely transferred to the seat of the child seat. Such a situation may cause that the child transported in a child seat to not feel comfortable during the journey.

The analysis of the values of the determined indicators showed that the type of road surface on which the tests were carried out had a decisive influence on the level of acceleration recorded on the seats of child seats. Driving on a gravel road turned out to be less comfortable than driving on an asphalt road. The least comfortable drive was the cobblestone road.

The effective rms acceleration values determined for the tested vehicle seats are higher than the adult comfort limit, which is 0.315 m/s^2^. This limit applies to adults, as mentioned, and it is difficult to determine the feelings of children.

The leg stabilization of the IQ ISOFIX base transmits the vibrations to the seat directly from the floor. Consequently, it contributes to the deterioration of the vibration comfort of children carried in the seat. The negative impact of separating the child seat from the rear seat of the vehicle using the IQ ISOFIX base is confirmed by the calculated indicators (rms, rmq and VDV). For the child seat fastened in a classic way, the values of these indicators were significantly lower than the values of the indicators determined for the seat fastened with the ISOFIX system.

There are no standards for carrying out tests on the vibration comfort of children. To better identify the issue of the propagation of vibrations in the child seat system it is desirable to conduct tests concerning more seats. Further research is necessary due to the need to develop standards for carrying out tests and assessing the vibration comfort of children. Additionally, guidelines should also be drawn up regarding the maximum length of time a child can be seated in the seat without negative consequences for their health.

Therefore, in the future, the child’s seat should be considered in addition to the standard sensors of puncture, blood pressure sensors or sensors of the correct fastening of the seat, and they should be equipped with an acceleration sensor that determines the level of vibrations from the road surface to the seat of the child’s seat. In order to reduce the level of acceleration recorded on child seats, the ISOfix base should be expanded with a damping element, with the task of dampening the transmitted vibrations.

This study, concerning the vibration comfort of children, should not discourage parents from transporting their children in vehicle seats, as they are the most effective product protecting children against injuries and deaths during road accidents.

## Figures and Tables

**Table 1:** Parameters of the tested child seats [61].

Parameter	Avionaut Pixel Child Seat	Avionaut AeroFIX Child Seat
Dimensions:		
height, cm	44	73
width, cm	58	43
depth, cm	70	66
Mass of the child seat, kg	2.5	4
Height of the transported child, cm	45–86	67–105
Maximum mass of the transported child, kg	to 13	to 17.5
Possibility to attach the child seat with the seat belts	Yes	Yes
Possibility to attach the child seat with the ISOFIX base, which is stabilized with a supporting leg	Yes	Yes
Forward-facing mounting possible	Yes	Yes
Rearward-facing mounting possible	Yes	Yes

**Table 2:** Technical parameters of acceleration sensors.

Parameter	Value
Maximum measuring range, m/s^2^	98
Frequency band measured, Hz	0.51000
Resonant frequency, Hz	≥27,000
Sensitivity, mV/(m/s^2^)	10.2
Number of axles	3

**Table 3:** The values of vibration comfort indicators.

Surface Type	Child Mass, kg	Comfort Rating Index	Avionaut Pixel Child Seat		Avionaut AeroFIX Child Seat	
			Acceleration Sensor Location			
			Vehicle Rear Seat	The Seat of the Avionaut Pixel Child Seat	Isofix Base	The Seat of the Avionaut AeroFIX Child Seat
asphalt road	5	rms, m/s^2^	0.314	0.396	0.354	0.617
		VDV, m/s^1.75^	1.258	1.682	1.491	2.468
		rqm, m/s^2^	0.452	0.604	0.536	0.887
	10	rms, m/s^2^	0.270	0.357	0.307	0.392
		VDV, m/s^1.75^	1.014	1.377	1.299	1.681
		rqm, m/s^2^	0.364	0.495	0.467	0.604
	15	rms, m/s^2^	0.269	0.340	0.312	0.396
		VDV, m/s^1.75^	1.027	1.408	1.615	1.986
		rqm, m/s^2^	0.369	0.506	0.580	0.713
gravel road	5	rms, m/s^2^	1.097	1.379	1.487	1.886
		VDV, m/s^1.75^	4.744	6.066	6.376	7.505
		rqm, m/s^2^	1.705	2.179	2.291	2.697
	10	rms, m/s^2^	1.136	1.474	1.418	1.753
		VDV, m/s^1.75^	4.580	6.210	5.786	7.116
		rqm, m/s^2^	1.645	2.231	2.079	2.557
	15	rms, m/s^2^	0.896	1.298	1.206	1.575
		VDV, m/s^1.75^	3.601	5.405	5.291	6.489
		rqm, m/s^2^	1.294	1.942	1.901	2.331
cobblestone road	5	rms, m/s^2^	2.452	2.336	2.862	3.556
		VDV, m/s^1.75^	9.512	8.973	12.445	13.867
		rqm, m/s^2^	3.418	3.224	4.471	4.982
	10	rms, m/s^2^	1.786	2.581	2.437	2.856
		VDV, m/s^1.75^	6.602	9.392	10.167	10.902
		rqm, m/s^2^	2.372	3.375	3.653	3.917
	15	rms, m/s^2^	1.514	2.184	2.338	2.689
		VDV, m/s^1.75^	5.599	7.988	9.289	10.088
		rqm, m/s^2^	2.012	2.870	3.337	3.624

## Data Availability

Not applicable.
